# Consideration of image guidance in patterns of failure analyses of intensity-modulated radiotherapy for head and neck cancer: a systematic review

**DOI:** 10.1186/s13014-024-02421-w

**Published:** 2024-03-05

**Authors:** Paul-Henry Mackeprang, Katarina Bryjova, Astrid E. Heusel, Dominik Henzen, Melissa Scricciolo, Olgun Elicin, on behalf of the EORTC Head & Neck Young Investigators Group

**Affiliations:** 1 Department of Radiation Oncology, Inselspital, Bern University Hospital and University of Bern, 3010 Bern, Switzerland; 2 Division of Medical Radiation Physics, Inselspital, Bern University Hospital and University of Bern, 3010 Bern, Switzerland; 3 Radiation Oncology Division, Clinical Radiology Department, Ospedale dell’Angelo, Via Paccagnella 11, 30174 Venezia, Italy

**Keywords:** Systematic review, Radiotherapy, Image-guided radiotherapy, Intensity-modulated radiotherapy, Patterns of failure, Head and neck, Squamous cell carcinoma, Follow-up studies, Recurrence, Treatment failure

## Abstract

**Background:**

Intensity-modulated radiation therapy (IMRT) is considered standard of care for head and neck squamous cell carcinoma (HNSCC). Improved conformity of IMRT and smaller margins, however, have led to concerns of increased rates of marginal failures. We hypothesize that while patterns of failure (PoF) after IMRT for HNSCC have been published before, the quality of patient positioning and image guided radiotherapy (IGRT) have rarely been taken into account, and their importance remains unclear. This work provides a systematic review of the consideration of IGRT in PoF studies after IMRT for HNSCC.

**Materials and methods:**

A systematic literature search according to PRISMA guidelines was performed on PubMed for HNSCC, IMRT and PoF terms and conference abstracts from ESTRO and ASTRO 2020 and 2021 were screened. Studies were included if they related PoF of HNSCC after IMRT to the treated volumes. Data on patient and treatment characteristics, IGRT, treatment adaptation, PoF and correlation of PoF to IGRT was extracted, categorized and analyzed.

**Results:**

One-hundred ten studies were included. The majority (70) did not report any information on IGRT. The remainder reported daily IGRT (18), daily on day 1–3 or 1–5, then weekly (7), at least weekly (12), or other schemes (3). Immobilization was performed with masks (78), non-invasive frames (4), or not reported (28). The most common PoF classification was “in-field/marginal/out-of-field”, reported by 76 studies. Only one study correlated PoF in nasopharyngeal cancer patients to IGRT.

**Conclusion:**

The impact of IGRT on PoF in HNSCC is severely underreported in existing literature. Only one study correlated PoF to IGRT measures and setup uncertainty. Further, most PoF studies relied on outdated terminology (“in/out-of-field”). A clearly defined and up-to-date PoF terminology is necessary to evaluate PoFs properly, as is systematic and preferably prospective data generation. PoF studies should consistently and comprehensively consider and report on IGRT.

**Supplementary Information:**

The online version contains supplementary material available at 10.1186/s13014-024-02421-w.

## Introduction

Head and neck cancer is one of the most common cancer types, being the seventh most frequent type worldwide, responsible for 3–4% of cancer cases and 1.5% of cancer deaths in the USA in 2018 [1]. The most common histological type is head and neck squamous cell carcinoma (HNSCC), occurring mostly in the oral or sino-nasal cavities, pharynx and larynx. Radiation therapy is one of the main pillars in the treatment of HNSCC as a definitive therapy in the early stages or combined with surgery and/or chemotherapy in advanced stages [2]. Despite advances in diagnostics and treatment techniques, recurrent and/or metastatic disease still develops in more than 65% of patients with HNSCC [1]. This shows the importance of the analysis of the patterns of failure (PoF) to identify potential treatment errors and thus opportunities for improvement.

Meta-analysis showed intensity-modulated radiation therapy (IMRT) for HNSCC to be associated with a lower risk for xerostomia compared to 3D-conformal radiation therapy (3DCRT) [3] and was therefore adopted as standard of care. IMRT allows for more conformal dose distributions to improve sparing of organs-at-risk (OARs). However, this raises concerns over geographical misses and under-dosing of the target due to treatment uncertainties and the close anatomical relationship between target volumes and OARs in HNSCC [4]. To ensure treatment success, several factors need to be considered. Beside other factors, such as correct dose/fractionation or accurate target volume definition, precise treatment delivery has paramount importance for oncologic outcome. This precision is ensured by image guidance (image-guided radiotherapy, IGRT), and residual uncertainty is mitigated by safety margins around the clinical target volume (CTV) [5]. Margins greatly increase the target volumes and thus contradict OAR sparing [6] leading to a compromise between the risk of local and regional failure and treatment toxicity [7]. IGRT is implemented to allow tighter treatment margins by increasing the accuracy and precision of patient setup.

To investigate this possibility, PoF studies assess the location of treatment failure in follow-up of patients after IMRT for HNSCC. In these studies, a cohort of patients with recurrent disease after treatment is identified and the location of failure is categorized. In traditionally used broad terms, recurrences are classified as local, regional or distant, depending on their anatomical region relative to the initial primary tumor site. This classification however does not account for the initial treatment and therefore does not convey the evidence needed to improve treatment and margin decisions. Newer classifications relate recurrent tumor to the treatment by fusing the initial planning-CT, on which the radiotherapy plan was generated, to follow-up imaging on which a failure was detected. Based on that, the recurrences can be categorized into ‘in-volume’, ‘marginal’ and ‘outside of the treated volume’. Marginal recurrences are then scrutinized for possible systematic or random errors in terms of accuracy and precision of the equipment, target volume delineation, adherence to international guidelines, plan quality etc. However, none or very few of the previously published studies consider the influence of daily image-guidance quality (i.e., IGRT) on the PoF. Therefore, the interpretation of the patterns of post-IMRT recurrence may be non-reliable if the daily IGRT component is not considered. Our hypothesis was that this issue is severely underreported in existing literature.

The aim of this work was to review the existing literature on PoF in HNSCC after IMRT and to extract data from such studies with a focus on IGRT and PoF. The primary endpoint was to identify studies, which investigated the impact of IGRT on PoF. The secondary endpoint was to categorize and report on the methodology used to report PoF and IGRT in the examined body of literature.

## Materials and methods

### Identification and screening

A systematic literature search was performed through PubMed. Publication dates were restricted to January 1st, 1990 (prior to the clinical use of IMRT) to July 1st, 2023. The search terms implemented using Boolean operators were:

“Head and Neck Neoplasms“[Mesh] AND (radiother* OR radiat* OR irradiat* OR proton) AND (“intensity-modulated” OR “intensity modulated” OR IMRT OR VMAT OR “volumetric modulated arc” OR tomotherapy OR MRI-linac OR stereotactic) AND (recurren* OR failure).

No language restriction was applied. Further, conference abstracts of the last two annual meetings (2020 & 2021) of the American Society for Radiation Oncology (ASTRO) and the European Society for Radiotherapy and Oncology (ESTRO) were screened manually and relevant references in articles assessed in full text (both review and original) were added to the matches if they were not yet included. We followed the Preferred Reporting Items for Systematic Reviews and Meta-Analyses (PRISMA) guidelines [8] to document the search strategy and selection processes.

### Inclusion criteria

Included studies reported on PoF after IMRT of HNSCC, including the oral cavity, sino-nasal sites, nasopharynx, oropharynx, hypopharynx and larynx or cancer of unknown primary of the head and neck. Studies reporting on non-IMRT external beam delivery techniques (e.g. 2D and 3D conformal radiotherapy CRT) were excluded. Only studies that aimed to correlate the PoF by comparing the loco-regional tumor recurrence in follow-up imaging to target volumes and/or the prescription isodose volume were included. Included studies were screened in full-text and data was entered manually into pre-defined and structured spreadsheets. Table 1 details all extracted information.

**Table 1:** Data extracted from included studies

Category	Items
Patient characteristics	Number of cases Anatomical sub-sites TNM stages Radiotherapy setting Systemic treatment
Treatment characteristics	Radiotherapy technique Boost technique CTV-PTV margin
IGRT	Number and frequency of image acquisition Use of no-action-levels Primary and complementary/secondary IGRT techniques Immobilization techniques Setup correction
Adaptive re-planning	Workflow for adaptation Triggers for adaptation
Patterns of failure	Diagnostic Modality for PoF PoF correlation with target volumes PoF terminology
Analysis of IGRT factors influencing PoF	Possible associations between IGRT procedure and PoF Plan robustness analysis

CTV: Clinical Target Volume; IGRT: Image-Guided Radiotherapy; PoF: Patterns of Failure; PTV: Planning Target Volume; TNM: TNM Classification of Malignant Tumours

## Results

### Search results

**Fig. 1:**
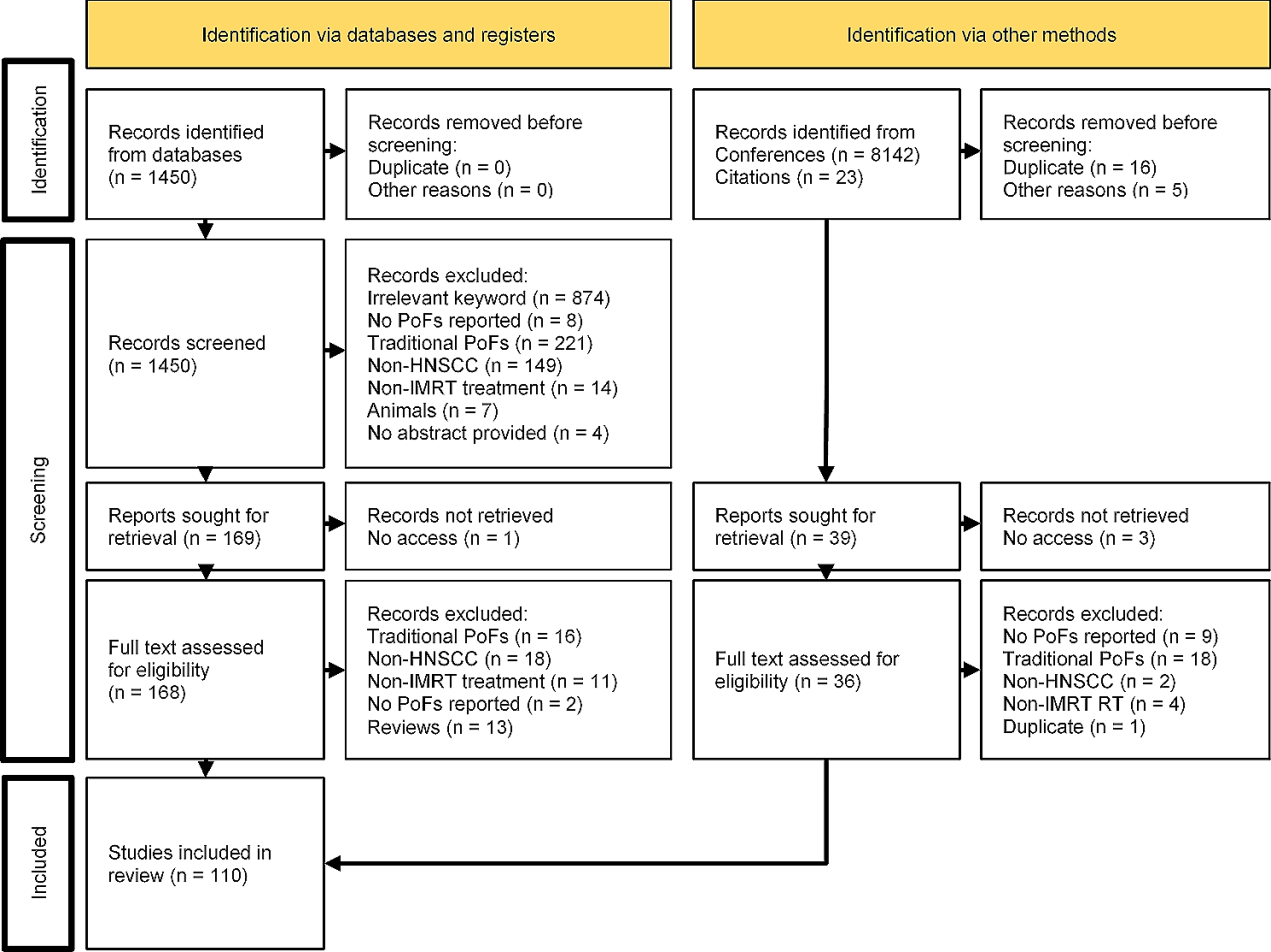
PRISMA flow diagram detailing numbers of screened, included and excluded articles and detailing data sources as well as reasons for exclusion of articles. HNSCC: Head-and-Neck Squamous Cell Carcinoma; PoF: Patterns of Failure

In total, 1450 articles were identified through PubMed and 39 through conference abstracts. Figure 1 shows the PRISMA [8] flow diagram with numbers of screened, included and excluded articles and reasons for eligibility. One-hundred ten studies were included in the final analysis, published between 1999 and 2023.

### Study cohorts and design

**Fig. 2:**
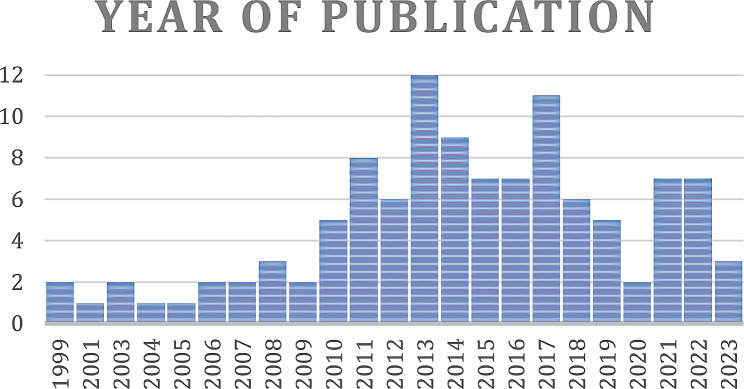
Publication year of included PoF studies. Interest in PoF studies increased from 2010 and continues to today. PoF: Patterns of Failure

Please see the supplementary material for all included references and a detailed breakdown of information reported by each study both in a per-item and per-study basis. In total, 110 studies were included (108 from PubMed, 2 from conference abstracts), reporting on 14,962 patients with an average of 136 (range 3–1576) patients per study. Oropharyngeal cancer was the most common anatomical tumor sub-site with 58 articles reporting results, followed by nasopharynx (57), larynx (48), oral cavity (45), hypopharynx (44), cancer of unknown primary of the head and neck (18) and sino-nasal sites (11). Included studies were published between 1999 and 2023 with a peak in 2013 as shown in Fig. 2. RT setting was primary in 61 studies, postoperative in 13 studies, mixed primary and postoperative in 24 studies, re-irradiation in 6 studies and not specified in 6 studies.

### Treatment characteristics

Systemic therapy was administered in most studies, with 97 studies reporting concomitant systemic therapy, 61 studies including patients that had RT alone, 52 studies including induction chemotherapy and 22 adjuvant. Only 5 studies did not report systemic treatment. Radiotherapy technique was specified as “IMRT” in 95 studies, with a minority adding or sub-specifying other techniques (VMAT: 14, Tomotherapy: 9, SBRT: 5, SBRT boost after IMRT: 4, brachytherapy boost: 2, IMPT: 2, orthovoltage boost: 1). In 25 studies, IMRT plans included conformal lower neck fields. In most studies, a simultaneous integrated boost technique (SIB) was used (89 studies), while 29 studies partially or exclusively included sequential treatments. Six studies performed single phase treatments and 5 studies did not report about the type of boost technique. CTV-PTV margins were generally between 3 and 5 mm, with only 11 studies deviating: Two studies did not use PTV margins, 4 studies used margins smaller than 3 mm, 4 studies allowed CTV-PTV margins of more than 5 mm (6, 7 and 8 mm) and one study used an anisotropic margin of 1 cm cranio-caudally and 5 mm in other directions. Twenty-four studies did not report CTV-PTV margins at all. Only 21 studies gave rationales for their choice of CTV-PTV margin and only 3 studies heuristically defined anisotropic margins for possible organ movement.

### Image guidance

The frequency of the imaging for set-up with or without couch correction was reported in 40 studies. Daily imaging was performed in 18 of them, daily on day 1–3 or 1–5, then weekly in 7, at least weekly imaging in 12, and other schemes in 3 studies. Only 1 study performed intrafraction IGRT with spine tracking during the boost phase. Throughout the studies reporting IGRT modalities, mostly a 3D volumetric imaging technique was used (21), followed by 2D (14). An unknown mix of modalities was used in 6 studies and one study used surface imaging as a secondary image guidance technique. The most frequent matching structure was bone (13). Only 3 studies used soft tissue for matching. The matching was checked online in 18 studies, offline and less frequently than each fraction in 8 studies. The degrees of freedom (DoF) for setup correction were reported in 10 studies with one of them explicitly specifying a 6DoF couch for rotational error correction, and the remainder using translations only (3DoF). Eight studies reported having set up a “no-action-level”, most commonly of 3 mm. The patient immobilization was reported in 83 studies. For immobilization of the head and shoulder region, head, neck and shoulder masks (39) were used most frequently or unspecified types of masks (29), head and neck (8) and head masks (2). Further, a bite block was used in 7 studies, and 17 studies used other techniques (mostly shoulder supports or vacuum-fixed cushions). Four older SBRT studies reported using stereotactic head frame fixation (either invasive or non-invasive) instead of masks and 28 studies did not report patient immobilization. Only 4 studies reported using a standardized protocol for plan adaptation with 6 further studies reporting that plan adaptation could be initiated by the treating physician. All other studies either ruled out plan adaptation or did not report on it. Table 2 shows the details on the IGRT techniques.

**Table 2:** Image-Guided Radiotherapy Techniques

Frequency	Daily	Weekly	Day 1–3 or 1–5 daily, then weekly	Other		N/R
	18	12	7	3		70
**No-action-level used**	Yes	No				N/R
	8	14				88
**IGRT technique**	3D	2D	Surface scanner	Other		N/R
	21	14	1	6		72
**Matching structures**	Bone	Soft Tissue	Fiducials	Surface	Other	N/R
	13	3	0	0	0	96
**Immobilization mask**	Head	Head & neck	Head, neck & shoulder	Unspecified type	None	N/R
	2	8	39	29	4	28
**Immobilization support**	Bite block	Other	Stereotactic head frame (invasive and non-invasive)		None	N/R
	7	13	4		15	71
**Setup correction**	3DoF	4DoF	6DoF			N/R
	9	0	1			100

DoF: Degrees of freedom; IGRT: Image-guided radiotherapy; N/R: Not reported;

### Patterns of failure analysis

In the analysis of the PoF, the most frequent diagnostic modality was the combination of CT, MRI or PET/CT (24) followed by CT or MRI (21), CT or PET/CT (16) and MRI or PET/CT (4). CT, MRI or PET/CT as sole modality were performed in 10, 6 and 4 studies respectively. Thirty articles reported having used an additional clinical diagnostic modality such as endoscopy, neck dissection and/or biopsy. The remaining 20 studies did not report the follow-up modality used for PoF analysis or did not specify the type of imaging used. Only one study correlated the IGRT to PoF, scrutinizing the set-up imaging in 15 patients with marginal recurrence of nasopharyngeal carcinoma. Analysis of set-up errors during IMRT treatment showed a high frequency of displacements exceeding 3 mm in one patient, which might have led to a marginal failure [9].

The image fusion used to specify the pattern of recurrence compared to the planning CT was in most cases rigid (40), whereas 12 studies used deformable fusion and 3 used both. Nine studies used anatomical or clinical correlation exclusively (2) or if imaging was not available (7) and the fusion technique was not reported in 53 studies. The most frequently used terms to describe PoF were „in-field“, „marginal“ and „out-of-field“, reported in slight variations by 76 of the 110 studies. All of the studies reported also on conventional PoF in terms of local, regional and distant failure. The plan robustness was mentioned in 8 studies, and not addressed in the remaining 102 studies. Two of these studies quantified robustness: The study of Zukauskaite et al. [10] performed a robustness evaluation by comparing the dose to 4 different possible points of origins (POs) of a recurrence (two independent observers, center of mass, maximum surface distance) and concluding that dose distributions were robust as dose between these points differed by less than 2%. The other study by Liu et al. assessed 10 patients with HNSCC that had 13 failures (9 in-field, 3 marginal, 1 out-of-field, confirmed by PET/CT) after being treated with VMAT. In this study, the authors performed a robustness analysis with assumed systematic setup shifts of 3 mm in all directions, derived from their choice of CTV-PTV margin, as they did not report IGRT specifics. They concluded, that 11 out of 13 failures were associated with an underdosage in the perturbed dose distribution, leading them to conclude that for the included patients, “the possible target underdosage due to patient setup uncertainties appeared to be a more relevant factor associated with local failure […] than the compromised PTV coverage” [11].

## Discussion

We extracted, categorized and analyzed data from 110 eligible studies on PoF in HNSCC after IMRT. The number of studies published on this topic increased from 2010 and the interest continues until this day. However, most of these studies did not correlate the influence of their IGRT quality to the PoF. In most cases, the IGRT technique was reported only briefly or not at all. Also, the PoF terms are reported and defined in many different ways throughout the included studies. Even in the recent publications, the most frequently used terms are “in-/out-of-*field”*, relying on outdated field-based terminology instead of volume or dosimetric definitions. An up-to-date and clear definition of in-*volume*, marginal and out-of-*volume* failures are needed to assess PoF correctly and analyze them properly and systematically.

Only one study correlated the IGRT component to the PoF in patients with loco-regionally recurrent nasopharyngeal carcinoma. In this study, for one patient the analysis of set-up uncertainties showed a systematic error of 1.0–1.5 mm and a random component with 20% of deviations > 3 mm. This was judged by the authors to be inadequate setup reproducibility, which contributed to the observed marginal failure. Setup uncertainty was further quantified for 14 other patients, but not judged to be the main cause of failure. We did not find any other publication performing this kind of analysis of the IGRT set-up and correlating it to the PoF. It is suggestive that this component may influence the recurrence patterns and rate in HNSCC after IMRT, however due to lack of data, it is unclear to what extent. Other similar reviews to our study have looked at PoF after IMRT for HNSCC, but have not mentioned or investigated the consideration of IGRT at all [12–14] or have addressed the issue by not providing any data or recommendations for improvement [15, 16]. One review [16] mentions the role of IGRT in the treatment and reports PoFs, however it does not examine the influence of the IGRT quality on them. The other does acknowledge “With the use of smaller and more precise treatment volumes, accurate target delineation, and tracking/monitoring during treatment is necessary to reduce the risk of a marginal miss” [15], but does not provide any review of IGRT influence on PoF. While the extent of this problem is thus not known, the impact of elevated setup uncertainty in IMRT for HNSCC has been examined by robustness studies [17], providing evidence of underdosage of target volumes and increased dose to OARs. Both endpoints have been linked to inferior oncologic outcomes and increased toxicity. Due to this “in-silico evidence”, guidelines recommend daily image guidance in HNSCC [18–21], and ICRU 83 providing its “best practice” examples with daily imaging for IMRT [22].

Our analysis of the literature and report is aimed at raising awareness of the insufficient evidence and consideration of IGRT in PoF studies. It is, however, not aimed at quantifying the influence of different IGRT patterns due to the restrictions of the published data. It therefore cannot be used to generate comprehensive evidence or to derive future guidelines on IGRT for IMRT for HNSCC. This will have to be left for future studies. The main limitation of the evidence we were able to generate from this review comes from the level of data that the underlying studies provide. First, the included studies used considerable heterogeneity of the PoF and IGRT terms and, in some cases, even different definitions for the same terms. Secondly, most included studies were of retrospective nature with its inherent limitations and biases. Lastly, most studies were published in the earlier days of IMRT, with some even using hybrid techniques with conformal lower-neck fields. Both the implementation of IMRT and IGRT evolved considerably in the meantime. Many aspects of the treatment have evolved since then, with new techniques, decreased target margins, more frequent and advanced image guidance and new delivery systems leading to increased precision.

These limitations of the underlying studies further support the main finding of this review of insufficient evidence on the topic and warrant the creation of systematically (and favorably prospectively) generated data to form future guidance. One of the key topics to assess will be the relevance of IGRT compared to other sources of uncertainty, such as target delineation, intrafraction motion, underlying tumor biology or other factors. Raising awareness on IGRT specifics is especially important as it can be assumed that most– at least most contemporary - studies did perform some form of image guidance but did not report on it. Especially, studies examining daily online adaptive IGRT may be able to provide separation between the influence of IGRT, target delineation and other sources of uncertainty as all these factors are intrinsically recorded in great detail as part of the study aim, if the quality and the robustness of the adaptive technique is ensured. This review is aimed to encourage all PoF study reports to include the specifics of IGRT and correlate them to the observed patterns.

To assess the extent of the impact of IGRT quality on the PoF in HNSCC, we need more, preferably prospective data on this issue. As a prerequisite, establishing a clearly defined and up to date PoF terminology is necessary to categorize and evaluate the data properly. Authors conducting PoF studies after IMRT for HNSCC should include both PoF terms correlated to the treated volumes and IGRT specifics used during treatment in every published series. Further, generation of prospective evidence examining the correlation between IMRT and PoF and identifying underlying factors is warranted.

## Conclusion

We were able to confirm that the influence of the quality of IGRT on the PoF in HNSCC is severely underreported in the available literature. Only one study correlated IGRT to PoF and definitions of PoF terms mostly rely on outdated terminology (“in/out-of-field”). Establishing a clearly defined and up-to-date PoF terminology is necessary to categorize and evaluate PoFs properly. Systematical, and preferably prospective, generation of data is warranted and needed to form the basis of future guidance. PoF studies should consistently and comprehensively consider and report on IGRT.

### Electronic supplementary material

Below is the link to the electronic supplementary material.

Supplementary Material 1

Supplementary Material 2

## Data Availability

The datasets used and/or analysed during the current study are available from the corresponding author on reasonable request.

## Abbreviations

3DoF: 3-Degrees of freedom (translations)
3D-CRT: 3D-Conformal Radiation Therapy
ASTRO: American Society for Radiation Oncology
CTV: Clinical Target Volume
DoF: Degrees of Freedom
ESTRO: European Society for Radiotherapy and Oncology
HNSCC: Head and Neck Squamous Cell Carcinoma
IGRT: Image-Guided Radiotherapy
IMPT: Intensity-Modulated Proton Therapy
IMRT: Intensity-Modulated Radiation Therapy
N/R: Not Reported
NST: No specific Type
OARs: Organs at Risk
PoF: Patterns of Failure
PRISMA: Preferred Reporting Items for Systematic Reviews and Meta-Analyses
PTV: Planning Target Volume
SBRT: Stereotactic Body Radiotherapy
SIB: Simultaneous Integrated Boost
VMAT: Volumetric Modulated Arc Therapy
