# Supplementary material for: Consideration of image guidance in patterns of failure analyses of intensity-modulated radiotherapy for head and neck cancer: a systematic review
Source: Radiat Oncol. 2024 Mar 5;19:30. doi: 10.1186/s13014-024-02421-w (PMC10916111; doi:10.1186/s13014-024-02421-w)
Supplement: Supplementary file 2 — Supplementary Material 2 [file 13014_2024_2421_MOESM2_ESM.docx]

Review Reference Table

| Statement | References No. |
| --- | --- |
| Included studies | 1 – 110 |
| From PubMed | 1 – 93, 96 – 110 |
| From conference abstracts | 94, 95 |
| Anatomical sub-site |  |
| Oropharynx | 1, 3, 4, 5, 6, 8, 13, 14, 15, 17, 18, 20, 21, 22, 24, 25, 26, 33, 34, 35, 36, 37, 38, 40, 42, 43, 44, 45, 48, 49, 53, 55, 58, 61, 62, 63, 64, 65, 69, 71, 72, 74, 75, 76, 77, 78, 79, 85, 86, 88, 90, 94, 96, 99, 101, 107, 109, 110 |
| Nasopharynx | 1, 2, 4, 7, 8, 9, 10, 17, 18, 21, 22, 26, 27, 28, 33, 35, 39, 41, 42, 43, 46, 49, 50, 51, 52, 53, 54, 56, 57, 62, 63, 66, 68, 70, 71, 73, 78, 79, 80, 81, 82, 83, 84, 86, 87, 89, 90, 91, 92, 93, 99, 100, 102, 103, 105, 106, 109 |
| Larynx | 1, 4, 6, 8, 12, 13, 15, 16, 17, 18, 21, 22, 25, 26, 33, 34, 35, 36, 38, 42, 43, 44, 45, 49, 53, 55, 61, 62, 63, 64, 65, 67, 69, 74, 76, 77, 78, 79, 85, 86, 88, 90, 94, 96, 101, 104, 109, 110 |
| Oral cavity | 1, 4, 6, 8, 11, 13, 17, 18, 19, 21, 22, 23, 25, 29, 30, 31, 32, 33, 34, 35, 36, 38, 42, 43, 44, 45, 47, 49, 53, 55, 59, 60, 61, 62, 69, 74, 75, 76, 78, 79, 85, 86, 88, 90, 109 |
| Hypopharynx | 1, 4, 6, 13, 15, 16, 18, 21, 22, 25, 26, 33, 34, 35, 36, 38, 42, 43, 44, 45, 49, 53, 55, 61, 62, 63, 64, 65, 67, 69, 71, 76, 77, 78, 79, 85, 86, 88, 90, 94, 96, 97, 101, 109 |
| Cancer of unknown primary of the head and neck | 4, 13, 15, 18, 21, 25, 33, 43, 45, 49, 64, 69, 71, 76, 78, 79, 86, 98 |
| Sino-nasal sites | 1, 8, 21, 33, 53, 78, 79, 86, 90, 99, 108 |
| RT setting |  |
| Primary | 2, 3, 5, 7, 9, 10, 12, 15, 18, 25, 26, 27, 28, 33, 36, 37, 39, 40, 41, 42, 46, 48, 50, 51, 52, 53, 54, 55, 56, 57, 58, 62, 64, 66, 67, 68, 70, 71, 72, 73, 74, 76, 77, 80, 81, 82, 83, 84, 87, 96, 97, 98, 101, 102, 103, 104, 105, 106, 107, 109, 110 |
| Postoperative | 11, 13, 19, 29, 30, 31, 32, 34, 38, 59, 60, 61, 75 |
| Mixed primary and postoperative or re-irradiation | 1, 4, 6, 8, 14, 16, 17, 20, 21, 23, 24, 35, 43, 45, 47, 49, 63, 65, 69, 78, 79, 85, 100, 108 |
| Re-irradiation | 86, 89, 91, 92, 93, 99 |
| Not specified | 22, 44, 88, 90, 94, 95 |
| Systemic therapy |  |
| Concomitant | 1, 2, 3, 4, 5, 6, 7, 8, 9, 10, 11, 13, 14, 15, 16, 17, 18, 19, 20, 21, 22, 23, 25, 26, 28, 29, 30, 31, 32, 33, 34, 35, 36, 37, 38, 39, 40, 41, 42, 43, 44, 45, 46, 47, 49, 50, 51, 52, 54, 55, 56, 57, 58, 59, 60, 61, 62, 63, 64, 65, 66, 67, 68, 69, 70, 71, 72, 73, 76, 77, 78, 79, 80, 81, 82, 83, 84, 85, 86, 87, 88, 90, 93, 95, 97, 98, 99, 100, 101, 102, 103, 105, 106, 107, 108, 109, 110 |
| RT alone | 7, 8, 11, 12, 16, 17, 38, 39, 40, 41, 42, 44, 45, 46, 47, 48, 49, 50, 52, 54, 55, 56, 58, 59, 60, 61, 62, 63, 64, 65, 68, 70, 71, 73, 75, 76, 78, 79, 80, 82, 83, 85, 86, 87, 88, 89, 90, 91, 92, 96, 97, 98, 99, 100, 102, 104, 105, 106, 108, 109, 110 |
| Induction | 1, 2, 4, 5, 6, 9, 10, 16, 18, 19, 20, 22, 25, 26, 27, 28, 33, 39, 40, 42, 46, 47, 50, 51, 52, 54, 57, 58, 66, 67, 68, 69, 70, 71, 73, 79, 80, 81, 82, 83, 84, 87, 90, 97, 99, 100, 102, 103, 105, 106, 107, 108 |
| Adjuvant | 2, 7, 10, 20, 21, 22, 23, 26, 27, 28, 33, 39, 41, 43, 50, 52, 56, 59, 70, 80, 105, 106 |
| Not reported | 24, 53, 74, 94, 95 |
| Radiotherapy technique |  |
| IMRT | 1, 2, 3, 4, 7, 8, 9, 10, 11, 12, 13, 14, 15, 16, 18, 19, 22, 23, 24, 25, 26, 27, 28, 29, 31, 32, 33, 34, 35, 36, 37, 38, 39, 40, 41, 42, 43, 44, 45, 46, 47, 48, 49, 50, 52, 53, 54, 55, 56, 57, 58, 59, 60, 61, 62, 63, 64, 65, 66, 67, 68, 69, 70, 71, 72, 73, 74, 75, 76, 77, 78, 79, 80, 81, 82, 83, 84, 87, 88, 90, 94, 95, 96, 97, 98, 100, 102, 103, 104, 105, 106, 107, 108, 109, 110 |
| IMRT plans included conformal lower neck fields. | 4, 7, 8, 9, 10, 14, 16, 26, 37, 40, 42, 45, 46, 52, 54, 58, 60, 65, 71, 75, 78, 79, 82, 83, 90 |
| Tomotherapy | 21, 29, 34, 43, 49, 51, 85, 88, 99 |
| VMAT | 5, 6, 17, 18, 30, 44, 55, 64, 94, 97, 101, 106, 108, 109 |
| SBRT | 86, 89, 91, 92, 93 |
| SBRT boost after IMRT | 3, 52, 68, 80 |
| brachytherapy boost | 4, 52 |
| IMPT | 20, 99 |
| Orthovoltage boost | 58 |
| Boost technique |  |
| Simultaneous integrated boost (SIB) | 1, 2, 4, 5, 6, 7, 8, 9, 10, 13, 14, 15, 16, 17, 18, 19, 20, 21, 22, 23, 25, 26, 27, 28, 29, 30, 31, 33, 34, 35, 36, 37, 38, 39, 40, 41, 42, 43, 44, 45, 46, 47, 48, 49, 50, 51, 52, 54, 55, 57, 58, 59, 61, 62, 64, 65, 66, 68, 70, 72, 74, 75, 76, 77, 78, 79, 80, 81, 82, 83, 84, 85, 87, 88, 90, 95, 96, 98, 99, 100, 101, 102, 103, 105, 106, 107, 108, 109, 110 |
| Sequential boost | 1, 3, 4, 6, 10, 11, 12, 30, 32, 36, 38, 46, 47, 51, 56, 57, 58, 63, 66, 67, 69, 72, 78, 79, 88, 94, 97, 105, 108 |
| Single phase treatments | 86, 89, 91, 92, 93, 104 |
| Not reported | 24, 53, 60, 71, 73 |
| CTV to PTV margin |  |
| No PTV margin | 78, 86 |
| < 3 mm | 68, 86, 103, 106 |
| more than 5 mm (6, 7 and 8 mm), | 42, 47, 79, 96 |
| anisotropic margin of 1 cm cranio-caudally and 5 mm in other directions. | 12 |
| Not reported | 9, 19, 20, 24, 31, 50, 52, 53, 60, 62, 64, 70, 74, 83, 89, 91, 92, 93, 94, 95, 98, 99, 100, 107 |
| Rationale given for choice of CTV-PTV margin | 7, 8, 12, 28, 29, 35, 37, 38, 40, 41, 42, 43, 45, 56, 72, 82, 86, 101, 102, 103, 104 |
| Heuristically defined anisotropic margins for possible organ movement. | 12, 48, 104 |
| IGRT frequency |  |
| Daily imaging | 10, 12, 15, 20, 21, 31, 33, 35, 49, 51, 67, 71, 73, 78, 86, 90, 97, 104 |
| Daily first to third or fifth fraction, then at least weekly | 1, 4, 30, 68, 72, 80, 96 |
| At least weekly | 3, 9, 14, 37, 39, 45, 62, 63, 66, 81, 101, 109 |
| Intrafraction IGRT with spine tracking during the boost phases. | 3 |
| Not reported | 2, 5, 6, 7, 8, 11, 13, 16, 17, 18, 19, 22, 23, 24, 25, 26, 27, 28, 32, 34, 36, 38, 40, 41, 42, 43, 44, 47, 48, 50, 52, 53, 54, 55, 56, 57, 58, 59, 60, 61, 64, 65, 69, 70, 74, 75, 76, 77, 79, 82, 83, 84, 85, 87, 88, 89, 91, 92, 94, 95, 98, 99, 100, 102, 103, 105, 106, 107, 108, 110 |
| IGRT technique |  |
| 3D volumetric imaging | 3, 12, 15, 20, 21, 30, 33, 34, 35, 39, 49, 51, 66, 73, 81, 90, 96, 97, 101, 104, 109 |
| 2D imaging | 1, 3, 4, 9, 10, 14, 15, 20, 22, 37, 45, 62, 63, 78 |
| Surface scanner | 12 |
| Unknown mix of modalities | 29, 31, 46, 68, 80, 86 |
| Not reported | 2, 5, 6, 7, 8, 11, 13, 16, 17, 18, 19, 23, 24, 25, 26, 27, 28, 32, 36, 38, 40, 41, 42, 43, 44, 47, 48, 50, 52, 53, 54, 55, 56, 57, 58, 59, 60, 61, 64, 65, 67, 69, 70, 71, 72, 74, 75, 76, 77, 79, 82, 83, 84, 85, 87, 88, 89, 91, 92, 93, 94, 95, 98, 99, 100, 102, 103, 105, 106, 107, 108, 110 |
| Matching structure |  |
| Bone | 1, 4, 9, 14, 21, 22, 29, 35, 37, 46, 49, 78, 90 |
| Soft tissue | 12, 46, 104 |
| Not reported | 2, 3, 5, 6, 7, 8, 10, 11, 13, 15, 16, 17, 18, 19, 20, 23, 24, 25, 26, 27, 28, 29, 30, 31, 32, 33, 34, 36, 38, 39, 40, 41, 42, 43, 44, 45, 47, 48, 50, 51, 52, 53, 54, 55, 56, 57, 58, 59, 60, 61, 62, 63, 64, 65, 66, 67, 68, 69, 70, 71, 72, 73, 74, 75, 76, 77, 79, 80, 81, 82, 83, 84, 85, 86, 87, 88, 89, 91, 92, 93, 94, 95, 96, 97, 98, 99, 100, 101, 102, 103, 105, 106, 107, 108, 109, 110 |
| Matching checks |  |
| Online | 1, 3, 4, 10, 12, 20, 21, 29, 30, 33, 35, 49, 67, 78, 86, 90, 96, 104 |
| Each fraction offline checked | 46 |
| Offline and less frequently than each fraction | 9, 14, 37, 39, 62, 63, 66 |
| Not reported | 2, 5, 6, 7, 8, 11, 13, 15, 16, 17, 18, 19, 22, 23, 24, 25, 26, 27, 28, 29, 31, 32, 34, 36, 38, 40, 41, 42, 43, 44, 45, 47, 48, 50, 51, 52, 53, 54, 55, 56, 57, 58, 59, 60, 61, 64, 65, 68, 69, 70, 71, 72, 73, 74, 75, 76, 77, 79, 80, 81, 82, 83, 84, 85, 87, 88, 89, 91, 92, 93, 94, 95, 97, 98, 99, 100, 101, 102, 103, 105, 106, 107, 108, 109, 110 |
| Setup correction |  |
| 6DoF | 97 |
| 3DoF | 21, 29, 30, 33, 46, 56, 72, 90, 104 |
| No-action level |  |
| No-action-level used | 1, 15, 30, 46, 63, 72, 96, 104 |
| Patient immobilization |  |
| Head, neck and shoulder mask | 1, 6, 10, 12, 14, 18, 20, 21, 23, 27, 28, 32, 33, 34, 35, 37, 38, 45, 46, 51, 52, 55, 56, 61, 62, 64, 67, 68, 71, 74, 75, 80, 81, 82, 85, 90, 104, 105, 108 |
| Unspecified type of mask | 3, 13, 16, 22, 30, 31, 36, 39, 41, 42, 43, 47, 48, 49, 54, 57, 59, 69, 70, 73, 76, 77, 79, 86, 87, 96, 99, 102, 109 |
| Head and neck mask | 9, 15, 25, 26, 63, 72, 84, 88 |
| Head masks | 66, 78 |
| Bite block | 20, 31, 32, 34, 73, 82, 90 |
| Other techniques (mostly shoulder supports or vacuum-fixed cushions). | 14, 23, 25, 26, 45, 55, 61, 66, 71, 78, 99, 105, 109 |
| Stereotactic head frame fixation (either invasive or non-invasive) | 89, 91, 92, 93 |
| Not reported | 2, 4, 5, 7, 8, 11, 17, 19, 24, 29, 40, 44, 50, 53, 58, 60, 65, 83, 94, 95, 97, 98, 100, 101, 103, 106, 107, 110 |
| Plan adaptation |  |
| Standardized workflow for adaptive re-planning in place | 3, 15, 56, 102 |
| No, but could be initiated by treating physician | 20, 37, 39, 49, 51, 66 |
| No or not reported | 1, 2, 4, 5, 6, 7, 8, 9, 10, 11, 12, 13, 14, 16, 17, 18, 19, 21, 22, 23, 24, 25, 26, 27, 28, 29, 30, 31, 32, 33, 34, 35, 36, 38, 40, 41, 42, 43, 44, 45, 46, 47, 48, 50, 52, 53, 54, 55, 57, 58, 59, 60, 61, 62, 63, 64, 65, 67, 68, 69, 70, 71, 72, 73, 74, 75, 76, 77, 78, 79, 80, 81, 82, 83, 84, 85, 86, 87, 88, 89, 90, 91, 92, 93, 94, 95, 96, 97, 98, 99, 100, 101, 103, 104, 105, 106, 107, 108, 109, 110 |
| Diagnostic modality for PoF analysis |  |
| CT, MRI or PET/CT | 1, 3, 5, 8, 11, 37, 41, 44, 46, 47, 55, 57, 69, 85, 87, 88, 97, 98, 99, 100, 102, 103, 108, 109 |
| CT or MRI | 2, 7, 13, 16, 28, 31, 32, 39, 45, 48, 56, 59, 66, 68, 72, 73, 76, 78, 80, 83, 93 |
| CT or PET/CT | 6, 15, 18, 19, 25, 33, 36, 42, 43, 60, 64, 65, 71, 77, 79, 110 |
| MRI or PET/CT | 24, 50, 51, 54 |
| CT alone | 12, 21, 26, 62, 74, 75, 86, 89, 90, 101 |
| MRI alone | 9, 10, 27, 52, 81, 82 |
| PET/CT alone | 17, 40, 95, 107 |
| Additional clinical diagnostic modalities | 3, 11, 36, 39, 40, 41, 44, 45, 48, 50, 52, 55, 56, 57, 64, 69, 77, 82, 92, 96, 98, 99, 100, 102, 103, 104, 105, 106, 108, 109 |
| PoF analysis not reported or unspecified type of imaging. | 4, 14, 20, 22, 23, 29, 30, 34, 35, 38, 49, 53, 58, 61, 63, 67, 70, 84, 91, 94 |
| IGRT correlated to PoF | 46 |
| Image fusion for PoF analysis |  |
| Rigid | 1, 3, 7, 8, 9, 10, 11, 12, 13, 14, 16, 17, 18, 23, 25, 26, 28, 30, 31, 33, 34, 37, 38, 40, 41, 42, 45, 46, 47, 48, 61, 69, 72, 75, 79, 81, 83, 85, 101, 106 |
| Deformable | 19, 20, 24, 43, 55, 62, 76, 86, 90, 95, 97, 109 |
| Both Rigid or Deformable | 44, 71, 74 |
| Anatomical or clinical correlation | 14, 23, 30, 42, 44, 69, 76, 99, 108 |
| Not reported | 2, 4, 5, 6, 15, 21, 22, 27, 29, 32, 35, 36, 39, 49, 50, 51, 52, 53, 54, 56, 57, 58, 59, 60, 63, 64, 65, 66, 67, 68, 70, 73, 77, 78, 80, 82, 84, 87, 88, 89, 91, 92, 93, 94, 96, 98, 100, 102, 103, 104, 105, 107, 110 |
| PoF terms |  |
| „In-field“, „marginal“ and „out-of-field“, reported in slight variation | 2, 3, 4, 6, 7, 8, 9, 10, 11, 12, 13, 14, 16, 17, 18, 20, 22, 23, 24, 25, 26, 28, 29, 30, 31, 32, 33, 34, 35, 38, 39, 40, 42, 43, 46, 50, 51, 52, 54, 57, 59, 60, 61, 65, 66, 68, 70, 72, 73, 76, 78, 80, 81, 82, 83, 84, 85, 86, 87, 88, 91, 92, 93, 94, 97, 99, 100, 101, 102, 103, 104, 105, 106, 108, 109, 110 |
| Plan robustness |  |
| Mentioned | 12, 20, 29, 33, 63, 71 |
| Quantified | 17, 55 |

# References

1. Bednarek C, Nguyen TVF, Puyraveau M, Bonnet, Lescut N, Azélie C, et al. Évaluation de la radiothérapie conformationnelle avec modulation d’intensité utilisée en pratique quotidienne pour les carcinomes de la tête et du cou. Cancer/Radiotherapie. 2017 Feb 1;21(1):21–7.

2. Liu F, Luo T, Jin T, Zhang J, Xiang Z, Yan R, et al. Advantages of using reduced-volume intensity modulated radiation therapy for the treatment of nasopharyngeal carcinoma: A retrospective paired study. BMC Cancer. 2019 Jun 8;19(1).

3. Baker S, Verduijn G, Petit S, Nuyttens JJ, Sewnaik A, van der Lugt A, et al. Locoregional failures and their relation to radiation fields following stereotactic body radiotherapy boost for oropharyngeal squamous cell carcinoma. Head Neck. 2019 Jun 1;41(6):1622–31.

4. Toledano I, Graff P, Serre A, Boisselier P, Bensadoun RJ, Ortholan C, et al. Intensity-modulated radiotherapy in head and neck cancer: Results of the prospective study GORTEC 2004-03. Radiother Oncol. 2012 Apr;103(1):57–62.

5. Franzese C, Fogliata A, Franceschini D, Clerici E, D’agostino G, Navarria P, et al. Treatment: Outcome and toxicity of volumetric modulated arc therapy in oropharyngeal carcinoma. Anticancer Res. 2016;36(7):3451–7.

6. Moncharmont C, Vallard A, Guy JB, Prades JM, Rancoule C, Magné N. Real-life efficacy of volumetric modulated arc therapy in head and neck squamous cell carcinoma. Eur Ann Otorhinolaryngol Head Neck Dis. 2017 May 1;134(3):165–9.

7. Li JG, Venigalla P, Leeman JE, LaPlant Q, Setton J, Sherman E, et al. Patterns of nodal failure after intensity modulated radiotherapy for nasopharyngeal carcinoma. Laryngoscope. 2017 Feb 1;127(2):377–82.

8. Johansen S, Norman MH, Dale E, Amdal CD, Furre T, Malinen E, et al. Patterns of local-regional recurrence after conformal and intensity-modulated radiotherapy for head and neck cancer. Radiat Oncol. 2017 May 25;12(1):1.

9. Li J xin, Huang S min, Jiang X hua, Ouyang B, Han F, Liu S, et al. Local failure patterns for patients with nasopharyngeal carcinoma after intensity-modulated radiotherapy. Radiat Oncol. 2014 Mar 27;9(1).

10. Ng WT, Lee MCH, Hung WM, Choi CW, Lee KC, Chan OSH, et al. Clinical outcomes and patterns of failure after intensity-modulated radiotherapy for nasopharyngeal carcinoma. Int J Radiat Oncol Biol Phys. 2011 Feb 1;79(2):420–8.

11. Lin YW, Chen YF, Yang CC, Ho CH, Wu TC, Yen CY, et al. Patterns of failure after postoperative intensity-modulated radiotherapy for locally advanced buccal cancer: Initial masticator space involvement is the key factor of recurrence. Head Neck. 2018 Dec 1;40(12):2621–32.

12. Rock K, Huang SH, Tiong A, Lu L, Xu W, Ringash J, et al. Partial Laryngeal IMRT for T2N0 Glottic Cancer: Impact of Image Guidance and Radiation Therapy Intensification. Int J Radiat Oncol Biol Phys. 2018 Nov 15;102(4):941–9.

13. Ooishi M, Motegi A, Kawashima M, Arahira S, Zenda S, Nakamura N, et al. Patterns of failure after postoperative intensity-modulated radiotherapy for locally advanced and recurrent head and neck cancer. Jpn J Clin Oncol. 2016 Oct 1;46(10):919–27.

14. Daly ME, Le QT, Maxim PG, Loo BW, Kaplan MJ, Fischbein NJ, et al. Intensity-Modulated Radiotherapy in the Treatment of Oropharyngeal Cancer: Clinical Outcomes and Patterns of Failure. Int J Radiat Oncol Biol Phys. 2010 Apr;76(5):1339–46.

15. Rumley CN, Nedev N, Sharples K, Lee J, Lamb DS. Intensity-modulated radiotherapy in the treatment of locoregionally advanced head and neck cancer: implementation and outcomes in a New Zealand community hospital. J Med Radiat Sci. 2016 Jun 1;63(2):96–103.

16. Daly ME, Le QT, Jain AK, Maxim PG, Hsu A, Loo BW, et al. Intensity-modulated radiotherapy for locally advanced cancers of the larynx and hypopharynx. Head Neck. 2011 Jan;33(1):103–11.

17. Liu W, Patel SH, Harrington DP, Hu Y, Ding X, Shen J, et al. Exploratory study of the association of volumetric modulated arc therapy (VMAT) plan robustness with local failure in head and neck cancer. J Appl Clin Med Phys. 2017 Jul 1;18(4):76–83.

18. Chajon E, Lafond C, Louvel G, Castelli J, Williaume D, Henry O, et al. Salivary gland-sparing other than parotid-sparing in definitive head-and-neck intensity-modulated radiotherapy does not seem to jeopardize local control. Radiat Oncol. 2013 May 30;8(1).

19. Mohamed ASR, Wong AJ, Fuller CD, Kamal M, Gunn GB, Phan J, et al. Patterns of locoregional failure following post-operative intensity-modulated radiotherapy to oral cavity cancer: Quantitative spatial and dosimetric analysis using a deformable image registration workflow. Radiat Oncol. 2017 Aug 15;12(1).

20. Gunn GB, Blanchard P, Garden AS, Zhu XR, Fuller CD, Mohamed AS, et al. Clinical Outcomes and Patterns of Disease Recurrence after Intensity Modulated Proton Therapy for Oropharyngeal Squamous Carcinoma. Int J Radiat Oncol Biol Phys. 2016 May 1;95(1):360–7.

21. Chen AM, Jennelle RLS, Sreeraman R, Yang CC, Liu T, Vijayakumar S, et al. Initial clinical experience with helical tomotherapy for head and neck cancer. Head Neck. 2009 Dec;31(12):1571–8.

22. Nangia S, Chufal KS, Arivazhagan V, Srinivas P, Tyagi A, Ghosh D. Compensator-based Intensity-modulated Radiotherapy in Head and Neck Cancer: Our Experience in Achieving Dosimetric Parameters and their Clinical Correlation. Clin Oncol. 2006 Aug;18(6):485–92.

23. Daly ME, Le QT, Kozak MM, Maxim PG, Murphy JD, Hsu A, et al. Intensity-modulated radiotherapy for oral cavity squamous cell carcinoma: Patterns of failure and predictors of local control. Int J Radiat Oncol Biol Phys. 2011 Aug 1;80(5):1412–22.

24. Chen AM, Chin R, Beron P, Yoshizaki T, Mikaeilian AG, Cao M. Inadequate target volume delineation and local–regional recurrence after intensity-modulated radiotherapy for human papillomavirus-positive oropharynx cancer: Local–regional recurrence after IMRT for HPV-positive oropharynx cancer. Radiother Oncol. 2017 Jun 1;123(3):412–8.

25. De Felice F, Thomas C, Barrington S, Pathmanathan A, Lei M, Urbano TG. Analysis of loco-regional failures in head and neck cancer after radical radiation therapy. Oral Oncol. 2015 Nov 1;51(11):1051–5.

26. Schoenfeld GO, Amdur RJ, Morris CG, Li JG, Hinerman RW, Mendenhall WM. Patterns of Failure and Toxicity after Intensity-Modulated Radiotherapy for Head and Neck Cancer. Int J Radiat Oncol Biol Phys. 2008 Jun 1;71(2):377–85.

27. Xue F, Hu C, He X. Impact of minimum point dose on local control and toxicity in T3-4 nasopharyngeal carcinoma treated with intensity-modulated radiation therapy plus chemotherapy. Jpn J Clin Oncol. 2018 Mar 1;48(3):265–71.

28. Kong F, Ying H, Du C, Huang S, Zhou J, Chen J, et al. Patterns of local-regional failure after primary intensity modulated radiotherapy for nasopharyngeal carcinoma. Radiat Oncol. 2014 Feb 19;9(1).

29. Hsieh CH, Shueng PW, Wang LY, Huang YC, Liao LJ, Lo WC, et al. Impact of postoperative daily image-guided intensity-modulated radiotherapy on overall and local progression-free survival in patients with oral cavity cancer. BMC Cancer. 2016 Feb 23;16(1).

30. Chakraborty S, M Patil V, Babu S, Muttath G, Thiagarajan SK. Locoregional recurrences after post-operative volumetric modulated arc radiotherapy (VMAT) in oral cavity cancers in a resource constrained setting: Experience and lessons learned. Br J Radiol. 2015 Apr 1;88(1048).

31. Chan AK, Huang SH, Le LW, Yu E, Dawson LA, Kim JJ, et al. Postoperative intensity-modulated radiotherapy following surgery for oral cavity squamous cell carcinoma: Patterns of failure. Oral Oncol. 2013 Mar;49(3):255–60.

32. Geretschläger A, Bojaxhiu B, Crowe S, Arnold A, Manser P, Hallermann W, et al. Outcome and patterns of failure after postoperative intensity modulated radiotherapy for locally advanced or high-risk oral cavity squamous cell carcinoma. Radiat Oncol. 2012 Oct 22;7(1).

33. Dandekar V, Morgan T, Turian J, Fidler MJ, Showel J, Nielsen T, et al. Patterns-of-failure after helical tomotherapy-based chemoradiotherapy for head and neck cancer: Implications for CTV margin, elective nodal dose and bilateral parotid sparing. Oral Oncol. 2014;50(5):520–6.

34. Stromberger C, Jann D, Becker ET, Raguse JD, Tinhofer I, Marnitz S, et al. Adjuvant simultaneous integrated boost IMRT for patients with intermediate- And high-risk head and neck cancer: Outcome, toxicities and patterns of failure. Oral Oncol. 2014;50(11):1114–21.

35. Chen AM, Farwell DG, Luu Q, Donald PJ, Perks J, Purdy JA. Evaluation of the planning target volume in the treatment of head and neck cancer with intensity-modulated radiotherapy: What is the appropriate expansion margin in the setting of daily image guidance? Int J Radiat Oncol Biol Phys. 2011 Nov 15;81(4):943–9.

36. McCloskey SA, Jaggernauth W, Rigual NR, Hicks WL, Popat SR, Sullivan M, et al. Radiation treatment interruptions greater than one week and low hemoglobin levels (12 g/dL) are predictors of local regional failure after definitive concurrent chemotherapy and intensity-modulated radiation therapy for squamous cell carcinoma of the head and neck. Am J Clin Oncol Cancer Clin Trials. 2009 Dec;32(6):587–91.

37. Huang K, Xia P, Chuang C, Weinberg V, Glastonbury CM, Eisele DW, et al. Intensity-modulated chemoradiation for treatment of stage III and IV oropharyngeal carcinoma: The University of California-San Francisco experience. Cancer. 2008 Aug 1;113(3):497–507.

38. Chen AM, Farwell DG, Luu Q, Chen LM, Vijayakumar S, Purdy JA. Misses and near-misses after postoperative radiation therapy for head and neck cancer: Comparison of IMRT and non-IMRT techniques in the CT-simulation era. Head Neck. 2010 Nov;32(11):1452–9.

39. Kawashima M, Ariji T, Kameoka S, Ueda T, Kohno R, Nishio T, et al. Locoregional control after intensity-modulated radiotherapy for nasopharyngeal carcinoma with an anatomy-based target definition. Jpn J Clin Oncol. 2013 Dec;43(12):1218–25.

40. Loo SW, Geropantas K, Wilson P, Martin WMC, Roques TW. Target Volume Definition for Intensity-modulated Radiotherapy after Induction Chemotherapy and Patterns of Treatment Failure after Sequential Chemoradiotherapy in Locoregionally Advanced Oropharyngeal Squamous Cell Carcinoma. Clin Oncol. 2013 Mar;25(3):162–70.

41. Wolden SL, Chen WC, Pfister DG, Kraus DH, Berry SL, Zelefsky MJ. Intensity-modulated radiation therapy (IMRT) for nasopharynx cancer: Update of the Memorial Sloan-Kettering experience. In: International Journal of Radiation Oncology Biology Physics. 2006. p. 57–62.

42. Caudell JJ, Meredith RF, Spencer SA, Keene KS, Dobelbower MC, Bonner JA. Margin on Gross Tumor Volume and Risk of Local Recurrence in Head-and-Neck Cancer. Int J Radiat Oncol Biol Phys. 2010 Jan;76(1):164–8.

43. Shakam A, Scrimger R, Liu D, Mohamed M, Parliament M, Field GC, et al. Dose-volume analysis of locoregional recurrences in head and neck IMRT, as determined by deformable registration: A prospective multi-institutional trial. Radiother Oncol. 2011 May;99(2):101–7.

44. Zukauskaite R, Hansen CR, Grau C, Samsøe E, Johansen J, Petersen JBB, et al. Local recurrences after curative IMRT for HNSCC: Effect of different GTV to high-dose CTV margins. Radiother Oncol. 2018 Jan 1;126(1):48–55.

45. Daly ME, Lieskovsky YY, Pawlicki T, Yau J, Pinto H, Kaplan M, et al. Evaluation of patterns of failure and subjective salivary function in patients treated with intensity modulated radiotherapy for head and neck squamous cell carcinoma. Head Neck. 2007 Mar;29(3):211–20.

46. Orlandi E, Tomatis S, Potepan P, Bossi P, Mongioj V, Carrara M, et al. Critical analysis of locoregional failures following intensity-modulated radiotherapy for nasopharyngeal carcinoma. Futur Oncol. 2013 Jan;9(1):103–14.

47. Yao M, Chang K, Funk GF, Lu H, Tan H, Wacha J, et al. The Failure Patterns of Oral Cavity Squamous Cell Carcinoma After Intensity-Modulated Radiotherapy-The University of Iowa Experience. Int J Radiat Oncol Biol Phys. 2007 Apr 1;67(5):1332–41.

48. Sanguineti G, Gunn GB, Endres EJ, Chaljub G, Cheruvu P, Parker B. Patterns of Locoregional Failure After Exclusive IMRT for Oropharyngeal Carcinoma. Int J Radiat Oncol Biol Phys. 2008 Nov 1;72(3):737–46.

49. Chen AM, Daly ME, Cui J, Mathai M, Benedict S, Purdy JA. Clinical outcomes among patients with head and neck cancer treated by intensity-modulated radiotherapy with and without adaptive replanning. Head Neck. 2014 Nov 1;36(11):1541–6.

50. Wang L, Guo Y, Xu J, Chen Z, Jiang X, Zhang L, et al. Clinical Analysis of Recurrence Patterns in Patients With Nasopharyngeal Carcinoma Treated With Intensity-Modulated Radiotherapy. Ann Otol Rhinol Laryngol. 2017 Dec 1;126(12):789–97.

51. Shueng PW, Shen BJ, Wu LJ, Liao LJ, Hsiao CH, Lin YC, et al. Concurrent image-guided intensity modulated radiotherapy and chemotherapy following neoadjuvant chemotherapy for locally advanced nasopharyngeal carcinoma. Radiat Oncol. 2011 Aug 13;6(1).

52. Zeng L, Sun XM, Chen CY, Han F, Huang Y, Xiao WW, et al. Comparative study on prophylactic irradiation to the whole neck and to the upper neck for patients with neck lymph node-negative nasopharyngeal carcinoma. Head Neck. 2014;36(5):687–93.

53. Sozio S, Rivera-Núñez Z, Mahmoud O, Kim S. Safety of differential radiation dosing in lymph node positive necks treated with IMRT. Pract Radiat Oncol. 2018 Jan 1;8(1):20–4.

54. Guo Q, Zheng Y, Lin J, Xu Y, Hu C, Zong J, et al. Modified reduced-volume intensity-modulated radiation therapy in non-metastatic nasopharyngeal carcinoma: A prospective observation series. Radiother Oncol. 2021 Mar 1;156:251–7.

55. Zukauskaite R, Hansen CR, Brink C, Johansen J, Asmussen JT, Grau C, et al. Analysis of CT-verified loco-regional recurrences after definitive IMRT for HNSCC using site of origin estimation methods. Acta Oncol (Madr). 2017 Nov 2;56(11):1554–61.

56. Nishimura Y, Shibata T, Nakamatsu K, Kanamori S, Koike R, Okubo M, et al. A Two-step intensity-modulated radiation therapy method for nasopharyngeal cancer: The Kinki University experience. Jpn J Clin Oncol. 2010 Feb;40(2):130–8.

57. Hu W, Zhu G, Guan X, Wang X, Hu C. The feasibility of omitting irradiation to the contralateral lower neck in stage N1 nasopharyngeal carcinoma patients. Radiat Oncol. 2013 Oct 4;8(1).

58. Garden AS, Dong L, Morrison WH, Stugis EM, Glisson BS, Frank SJ, et al. Patterns of disease recurrence following treatment of oropharyngeal cancer with intensity modulated radiation therapy. Int J Radiat Oncol Biol Phys. 2013 Mar 15;85(4):941–7.

59. Liu SH, Chao KSC, Leu YS, Lee JC, Liu CJ, Huang YC, et al. Guideline and preliminary clinical practice results for dose specification and target delineation for postoperative radiotherapy for oral cavity cancer. Head Neck. 2015 Jul 1;37(7):933–9.

60. Damast S, Wolden S, Lee N. Marginal recurrences after selective targeting with intensity-modulated radiotherapy for oral tongue cancer. Head Neck. 2012 Jun;34(6):900–6.

61. Chen AM, Farwell DG, Luu Q, Chen LM, Vijayakumar S, Purdy JA. Marginal misses after postoperative intensity-modulated radiotherapy for head and neck cancer. Int J Radiat Oncol Biol Phys. 2011 Aug 1;80(5):1423–9.

62. Due AK, Vogelius IR, Aznar MC, Bentzen SM, Berthelsen AK, Korreman SS, et al. Recurrences after intensity modulated radiotherapy for head and neck squamous cell carcinoma more likely to originate from regions with high baseline [18F]-FDG uptake. Radiother Oncol. 2014;111(3):360–5.

63. Collan J, Kapanen M, Mäkitie A, Nyman H, Joensuu H, Tenhunen M, et al. Submandibular gland-sparing intensity modulated radiotherapy in the treatment of head and neck cancer: Sites of locoregional relapse and survival. Acta Oncol (Madr). 2012 Jul;51(6):735–42.

64. Studer G, Huber GF, Holz E, Glanzmann C. Less may be more: nodal treatment in neck positive head neck cancer patients. Eur Arch Oto-Rhino-Laryngology. 2016 Jun 1;273(6):1549–56.

65. Gupta T, Jain S, Agarwal JP, Ghosh-Laskar S, Phurailatpam R, Pai-Shetty R, et al. Prospective assessment of patterns of failure after high-precision definitive (chemo)radiation in head-and-neck squamous cell carcinoma. Int J Radiat Oncol Biol Phys. 2011 Jun 1;80(2):522–31.

66. Saleh-Ebrahimi L, Zwicker F, Muenter MW, Bischof M, Lindel K, Debus J, et al. Intensity modulated radiotherapy (IMRT) combined with concurrent but not adjuvant chemotherapy in primary nasopharyngeal cancer - a retrospective single center analysis. Radiat Oncol. 2013 Jan 24;8(1).

67. Geretschläger A, Bojaxhiu B, Dal Pra A, Leiser D, Schmücking M, Arnold A, et al. Definitive intensity modulated radiotherapy in locally advanced hypopharygeal and laryngeal squamous cell carcinoma: Mature treatment results and patterns of locoregional failure. Radiat Oncol. 2015 Jan 17;10(1).

68. Cao C, Jiang F, Jin Q, Jin T, Huang S, Hu Q, et al. Locoregional extension and patterns of failure for nasopharyngeal carcinoma with intracranial extension. Oral Oncol. 2018 Apr 1;79:27–32.

69. Yossi S, El Alouani C, Pointreau Y, Laccourreye L, Capitain O, Gustin P, et al. Recurrence sites following definitive intensity-modulated conformational radiotherapy of squamous-cell carcinomas of the upper aerodigestive tract. Cancer/Radiotherapie. 2015 Apr 1;19(2):73–81.

70. Tang LL, Tang X ran, Li W fei, Chen L, Tian L, Lin AH, et al. The feasibility of contralateral lower neck sparing intensity modulation radiated therapy for nasopharyngeal carcinoma patients with unilateral cervical lymph node involvement. Oral Oncol. 2017 Jun 1;69:68–73.

71. Mohamed ASR, Rosenthal DI, Awan MJ, Garden AS, Kocak-Uzel E, Belal AM, et al. Methodology for analysis and reporting patterns of failure in the Era of IMRT: Head and neck cancer applications. Radiat Oncol. 2016 Jul 26;11(1):1.

72. Raktoe SAS, Dehnad H, Raaijmakers CPJ, Braunius W, Terhaard CHJ. Origin of tumor recurrence after intensity modulated radiation therapy for oropharyngeal squamous cell carcinoma. Int J Radiat Oncol Biol Phys. 2013 Jan 1;85(1):136–41.

73. Sanford NN, Lau J, Lam MB, Juliano AF, Adams JA, Goldberg SI, et al. Individualization of Clinical Target Volume Delineation Based on Stepwise Spread of Nasopharyngeal Carcinoma: Outcome of More Than a Decade of Clinical Experience. Int J Radiat Oncol Biol Phys. 2019 Mar 1;103(3):654–68.

74. Due AK, Vogelius IR, Aznar MC, Bentzen SM, Berthelsen AK, Korreman SS, et al. Methods for estimating the site of origin of locoregional recurrence in head and neck squamous cell carcinoma. Strahlentherapie und Onkol. 2012 Aug;188(8):671–6.

75. Wang ZH, Yan C, Zhang ZY, Zhang CP, Hu HS, Tu WY, et al. Outcomes and xerostomia after postoperative radiotherapy for oral and oropharyngeal carcinoma. Head Neck. 2014;36(10):1467–73.

76. Bayman E, Prestwich RJD, Speight R, Aspin L, Garratt L, Wilson S, et al. Patterns of Failure after Intensity-modulated Radiotherapy in Head and Neck Squamous Cell Carcinoma using Compartmental Clinical Target Volume Delineation. Clin Oncol. 2014;26(10):636–42.

77. Song JH o., Jeong BK, Choi HS, Jeong H, Kang MH e., Kang JH u., et al. Comparison of Failure Patterns Between Conventional and Intensity-modulated Radiotherapy for Stage III and IV Head and Neck Squamous Cell Carcinoma. Anticancer Res. 2015 Dec 1;35(12):6833–40.

78. Chao KSC, Ozyigit G, Tran BN, Cengiz M, Dempsey JF, Low DA. Patterns of failure in patients receiving definitive and postoperative IMRT for head-and-neck cancer. Int J Radiat Oncol Biol Phys. 2003 Feb 1;55(2):312–21.

79. Yao M, Dornfeld KJ, Buatti JM, Skwarchuk M, Tan H, Nguyen T, et al. Intensity-modulated radiation treatment for head-and-neck squamous cell carcinoma - The University of Iowa experience. Int J Radiat Oncol Biol Phys. 2005 Oct 1;63(2):410–21.

80. Cao CN, Luo JW, Gao L, Yi JL, Huang XD, Wang K, et al. Clinical outcomes and patterns of failure after intensity-modulated radiotherapy for T4 nasopharyngeal carcinoma. Oral Oncol. 2013 Feb;49(2):175–81.

81. Chen JLY, Huang YS, Kuo SH, Chen YF, Hong RL, Ko JY, et al. Intensity-modulated radiation therapy for T4 nasopharyngeal carcinoma: Treatment results and locoregional recurrence. Strahlentherapie und Onkol. 2013;189(12):1001–8.

82. Kam MKM, Teo PML, Chau RMC, Cheung KY, Choi PHK, Kwan WH, et al. Treatment of nasopharyngeal carcinoma with intensity-modulated radiotherapy: The Hong Kong experience. Int J Radiat Oncol Biol Phys. 2004 Dec 1;60(5):1440–50.

83. Chen JZ, Le QT, Han F, Lu LX, Huang SM, Lin CG, et al. Results of a phase 2 study examining the effects of omitting elective neck irradiation to nodal levels IV and Vb in patients with N 0-1 nasopharyngeal carcinoma. Int J Radiat Oncol Biol Phys. 2013 Mar 15;85(4):929–34.

84. Wang L, Wu Z, Xie D, Zeng R, Cheng W, Hu J, et al. Reduction of target volume and the corresponding dose for the tumor regression field after induction chemotherapy in locoregionally advanced nasopharyngeal carcinoma. Cancer Res Treat. 2019;51(2):685–95.

85. Farrag A, Voordeckers M, Tournel K, De Coninck P, Storme G. Pattern of failure after helical tomotherapy in head and neck cancer. Strahlentherapie und Onkol. 2010 Sep;186(9):511–6.

86. Wang K, Heron DE, Flickinger JC, Rwigema JCM, Ferris RL, Kubicek GJ, et al. A retrospective, deformable registration analysis of the impact of PET-CT planning on patterns of failure in stereotactic body radiation therapy for recurrent head and neck cancer. Vol. 4, Head and Neck Oncology. 2012.

87. Xiao F, Dou S, Li Y, Qian W, Liang F, Kong L, et al. Omitting the lower neck and sparing the glottic larynx in node-negative nasopharyngeal carcinoma was safe and feasible, and improved patient-reported voice outcomes. Clin Transl Oncol. 2019 Jun 14;21(6):781–9.

88. Leclerc M, Lartigau E, Lacornerie T, Daisne JF, Kramar A, Grégoire V. Primary tumor delineation based on 18FDG PET for locally advanced head and neck cancer treated by chemo-radiotherapy. Radiother Oncol. 2015 Jul 1;116(1):87–93.

89. Chua DTT, Sham JST, Hung KN, Leung LHT, Cheng PW, Kwong PWK. Salvage treatment for persistent and recurrent T1-2 nasopharyngeal carcinoma by stereotactic radiosurgery. Head Neck. 2001;23(9):791–8.

90. Mohamed ASR, Cardenas CE, Garden AS, Awan MJ, Rock CD, Westergaard SA, et al. Patterns-of-failure guided biological target volume definition for head and neck cancer patients: FDG-PET and dosimetric analysis of dose escalation candidate subregions. Radiother Oncol. 2017 Aug 1;124(2):248–55.

91. Chua DTT, Sham JST, Kwong PWK, Hung KN, Leung LHT. Linear accelerator-based stereotactic radiosurgery for limited, locally persistent, and recurrent nasopharyngeal carcinoma: Efficacy and complications. Int J Radiat Oncol Biol Phys. 2003 May 1;56(1):177–83.

92. Chua DTT, Sham JST, Hung KN, Kwong DLW, Kwong PWK, Leung LHT. Stereotactic radiosurgery as a salvage treatment for locally persistent and recurrent nasopharyngeal carcinoma. Head Neck. 1999;21(7):620–6.

93. Ahn YC, Kim DY, Huh SJ, Baek CH, Park K. Fractionated stereotactic radiation therapy for locally recurrent nasopharynx cancer: Report of three cases. Head Neck. 1999 Jul;21(4):338–45.

94. Laranja CADN, Rodrigues I, Ramos T, Castro B, Sousa F, Moreira D, et al. PO-0841: Treatment failure patterns after radical radiotherapy using modern radiation techniques. Radiother Oncol [Internet]. 2020 Nov 1;152:S453–4. Available from: http://www.thegreenjournal.com/article/S0167814021008586/fulltext

95. Basu T, Patineedi B, Vyaas H, Kendre P, Karpe A, Raut N, et al. PD-0660: Radical chemoradiation with IMRT-SIB for LAHNSCC: Preliminary outcome. Radiother Oncol. 2020 Nov 1;152:S366–7.

96. Pratap Singh N, Khurana R, Sapru S, Rastogi M, Gandhi AK, Rath S, et al. Long term outcome and late toxicity Of SIB-IMRT in definitive management of head and neck cancers in patients not suitable for chemo-radiotherapy. J Cancer Res Ther [Internet]. 2022 Oct 1;18(6):1461–8.

97. Otsuka M, Yasuda K, Uchinami Y, Tsushima N, Suzuki T, Kano S, et al. Detailed analysis of failure patterns using deformable image registration in hypopharyngeal cancer patients treated with sequential boost intensity-modulated radiotherapy. J Med Imaging Radiat Oncol [Internet]. 2023 Feb 1;67(1):98–110.

98. Ghatasheh H, Huang SH, Su J, Xu W, Bratman S V., Cho J, et al. Evaluation of risk-tailored individualized selection of radiation therapy target volume for head and neck carcinoma of unknown primary. Radiother Oncol [Internet]. 2022 Oct 1;175:56–64.

99. Beddok A, Saint-Martin C, Krhili S, Eddine CA, Champion L, Chilles A, et al. Curative high-dose reirradiation for patients with recurrent head and neck squamous cell carcinoma using IMRT or proton therapy: Outcomes and analysis of patterns of failure. Head Neck [Internet]. 2022 Nov 1;44(11):2452–64.

100. Xiao XT, Wu YS, Chen YP, Liu X, Guo R, Tang LL, et al. Patterns and prognosis of regional recurrence in nasopharyngeal carcinoma after intensity-modulated radiotherapy. Cancer Med [Internet]. 2023 Jan 1;12(2):1399–408. 101. Bicakci BC, Mustafayev TZ, Cetinayak O, Igdem S, Birgi SD, Meydan D, et al. Outcomes of carotid sparing intensity-modulated radiotherapy for early stage glottic cancer in 201 patients: Multicenter study of Turkish Radiation Oncology Society/TROD-01-007. Head Neck [Internet]. 2022 Aug 1;44(8):1825–32.

102. Xie DH, Wu Z, Li WZ, Cheng WQ, Tao YL, Wang L, et al. Individualized clinical target volume delineation and efficacy analysis in unilateral nasopharyngeal carcinoma treated with intensity-modulated radiotherapy (IMRT): 10-year summary. J Cancer Res Clin Oncol [Internet]. 2022 Aug 1;148(8):1931–42.

103. Choi WYL, Lai JWY, Yu ELM, Choy YH, Lam YN, Wong RKY, et al. Induction chemotherapy followed by radical chemoradiotherapy for patients with stage IV non-metastatic nasopharyngeal carcinoma: 11-Year Experience in a tertiary centre. J Med Imaging Radiat Oncol [Internet]. 2022 Sep 1;66(6):853–65.

104. Tans L, Al-Mamgani A, Kwa SLS, Elbers JBW, Keskin-Cambay F, Sewnaik A, et al. Single vocal cord irradiation for early-stage glottic cancer: Excellent local control and favorable toxicity profile. Oral Oncol [Internet]. 2022 Apr 1;127.

105. Liu X, Wu B, Huang J, Qin Y, Zhang Z, Shi L, et al. Tumor factors associated with in-field failure for nasopharyngeal carcinoma after intensity-modulated radiotherapy. Head Neck [Internet]. 2022 Apr 1;44(4):876–88.

106. Liao W, He J, Liu Z, Tian M, Yang J, Han J, et al. A novel dosimetric metrics-based risk model to predict local recurrence in nasopharyngeal carcinoma patients treated with intensity-modulated radiation therapy. Radiat Oncol [Internet]. 2021 Dec 1;16(1).

107. Klausner G, Troussier I, Kreps S, Fabiano E, Laccourreye O, Giraud P. [Impact of neck dissection in N2-3 oropharyngeal squamous cell carcinomas treated with definitive chemoradiotherapy: An observational real-life study]. Cancer Radiother [Internet]. 2021 Dec 1;25(8):771–8.

108. Slevin F, Pan S, Mistry H, Denholm M, Shor D, Oong Z, et al. A Multicentre UK Study of Outcomes for Locally Advanced Sinonasal Squamous Cell Carcinoma Treated with Adjuvant or Definitive Intensity-modulated Radiotherapy. Clin Oncol (R Coll Radiol) [Internet]. 2021 Oct 1;33(10):e450–61.

109. Pisani C, Vigna L, Mastroleo F, Loi G, Amisano V, Masini L, et al. Correlation of [18F] FDG-PET/CT with dosimetry data: recurrence pattern after radiotherapy for head and neck carcinoma. Radiat Oncol [Internet]. 2021 Dec 1;16(1).

110. Sher DJ, Pham NL, Shah JL, Sen N, Williams KA, Subramaniam RM, et al. Prospective Phase 2 Study of Radiation Therapy Dose and Volume De-escalation for Elective Neck Treatment of Oropharyngeal and Laryngeal Cancer. Int J Radiat Oncol Biol Phys [Internet]. 2021 Mar 15;109(4):932–40.
